# Association of Visceral Adiposity and Sarcopenia with Geospatial Analysis and Outcomes in Acute Pancreatitis

**DOI:** 10.3390/jcm14093005

**Published:** 2025-04-26

**Authors:** Ankit Chhoda, Manisha Bohara, Anabel Liyen Cartelle, Matthew Antony Manoj, Marco A. Noriega, Miriam Olivares, Jill Kelly, Olga Brook, Steven D. Freedman, Abraham F. Bezuidenhout, Sunil G. Sheth

**Affiliations:** 1 Division of Gastroenterology, Department of Medicine, Beth Israel Deaconess Medical Center, Boston, MA 02215, USA; ankitchhoda@gmail.com (A.C.); aliyenca@bidmc.harvard.edu (A.L.C.); mmanoj1@bidmc.harvard.edu (M.A.M.); mnorieg1@bidmc.harvard.edu (M.A.N.); sfreedma@bidmc.harvard.edu (S.D.F.); 2 Department of Radiology, Beth Israel Deaconess Medical Center, Boston, MA 02215, USA; manishabohara07@gmail.com (M.B.); obrook@bidmc.harvard.edu (O.B.); abezuide@bidmc.harvard.edu (A.F.B.); 3 Geographical Information System Library, Yale University, New Haven, CT 06520, USA; miriam.olivares@yale.edu (M.O.); jill.kelly@yale.edu (J.K.)

**Keywords:** visceral adiposity, acute pancreatitis, sarcopenia, social vulnerability index

## Abstract

**Background:** Radiological imaging has improved our insight into how obesity and sarcopenia impacts acute pancreatitis via several measured variables. However, we lack understanding of the association between social determinants of health and these variables within the acute pancreatitis population. **Methods:** This study included patients at a single tertiary care center between 1 January 2008 and 31 December 2021. Measurements of visceral adiposity (VA), subcutaneous adiposity (SA), the ratio of visceral to total adiposity (VA/TA), and degree of sarcopenia via psoas muscle Hounsfield unit average calculation (HUAC) were obtained on CT scans performed at presentation. Using geocoded patient data, we calculated the social vulnerability index (SVI) from CDC metrics. Descriptive and regression analyses were performed utilizing clinical and radiological data. **Results:** In 484 patients with 592 acute pancreatitis-related hospitalization, median (IQR) VA was 176 (100–251), SA was 209.5 (138.5–307), VA/TA ratio was 43.5 (32.3–55.3), and HUAC was 51.3 (44.4–58.9). For our primary outcome, geospatial analyses showed a reverse association between VA and SVI with a coefficient of −9.0 (*p* = 0.04) after adjustment for age, health care behaviors (i.e., active smoking and drinking), and CCI, suggesting residence in areas with higher SVI is linked to lower VA. However, VA/TA, SA, and HUAC showed no significant association with SVI. The SVI subdomain of socioeconomic status had significant association with VA (−39.78 (95% CI: −75.88–−3.70), *p* = 0.03) after adjustments. For our secondary outcome, acute pancreatitis severity had significant association with higher VA (*p* ≤ 0.001), VA/TA (*p* ≤ 0.001), and lower HUAC (*p* ≤ 0.001). When comparing single vs. recurrent hospitalization patients, there was significantly higher median VA with recurrences (VA-single acute pancreatitis: 149 (77.4–233) vs. VA-recurrent acute pancreatitis: 177 (108–256); *p* = 0.04). **Conclusions:** In this study we found that patients residing in more socially vulnerable areas had lower visceral adiposity. This paradoxical result potentially conferred a protective effect against severe and recurrent acute pancreatitis; however, this was not found to be statistically significant.

## 1. Introduction

Acute pancreatitis imposes significant clinical and economic challenges due to lengthy hospital stays and high healthcare costs [1]. Its severity ranges from mild to potentially fatal, with obesity significantly associated with worse outcomes [2,3]. The concurrent rise in the prevalence of both obesity and acute pancreatitis presents a major challenge to healthcare [4,5].

Body mass index (BMI) is a common measure of obesity, but it has limitations in differentiating between adipose tissue and muscle mass, as well as between various types of adipose tissue [6]. Visceral fat can exacerbate inflammation in acute pancreatitis, leading to the release of inflammatory and lipotoxic mediators that may trigger systemic inflammatory response syndrome (SIRS) [7,8]. Although anthropometric measures like waist circumference and waist-to-hip ratio provide indirect assessments of visceral fat, they do not accurately quantify its burden [9]. Imaging techniques, such as computed tomography (CT) scans, offer more precise indices of visceral adiposity (VA), subcutaneous adiposity (SA), and muscle mass. These radiologically quantifiable patterns or radiological features have been correlated with acute pancreatitis severity and its complications [10].

Additionally, several studies also demonstrated that sarcopenia is a poor prognostic factor in patients with acute pancreatitis. Sarcopenic obesity has been observed as highly prevalent in patients with severe acute pancreatitis and is a significant predictor of mortality [11]. Single axial CT images provide a reliable and accurate estimate of overall body composition. In a prior study comparing two methods (segmentation of all muscles on an axial image at L3 versus isolated psoas muscle measurement at L4) in patients with soft-tissue sarcoma, psoas Hounsfeld units and cross-sectional area (CSA) demonstrated a stronger predictive value for major surgical complications and mortality [12,13].

Despite the progress in understanding how obesity and sarcopenia affect acute pancreatitis, the influence of social determinants of health on these factors in this patient population remains under-explored. Factors such as socioeconomic status, access to healthcare, and environmental conditions can significantly impact health outcomes. These geospatial social determinants of health have been studied in conditions like acute myocardial infarction, trauma, and respiratory failure [14,15]. Geospatial analysis involves mapping regions to identify disparities in health outcomes and access to healthcare based on environmental factors. This analysis utilizes the Centers for Disease Control and Prevention Social Vulnerability Index (CDC-SVI), which evaluates 16 attributes across four sub-domains: socioeconomic status, household composition and disability, minority status and language, and housing type and transportation [16]. SVI can be applied across different levels, such as counties, ZIP codes, and census tracts, allowing for granular analysis of social vulnerability and its impact on health outcomes. Furthermore, based on physical address, SVI can be examined to understand the relationship of these social attributes with individual-level clinicoradiologic features and outcomes in acute pancreatitis [14]. Our study aimed to investigate geospatial social attributes which constitute SVI with the radiological features and clinical outcomes in patients with acute pancreatitis who presented to our medical center.

## 2. Materials and Methods

### 2.1. Study Design and Setting

We conducted a retrospective, observational study of patients diagnosed with acute pancreatitis and hospitalized at a single tertiary care center in Boston, Massachusetts between 1 January 2008, and 31 December 2021. This study was approved by the Institutional Review Board (Protocol ID: 2018P000613, IRB Approval Date 26/09/2024) with a waiver of informed consent, and reported per the Strengthening the Reporting of Observational Studies in Epidemiology (STROBE) reporting guideline [17].

### 2.2. Study Population

Adult patients (age ≥ 18 years) with acute pancreatitis were identified through electronic health record (EHR) based query for International Classification of Diseases, Ninth and Tenth Revision codes—577.0 and K85.9—and then manually screened to confirm the diagnosis based on the Revised Atlanta classification requiring two of the following three criteria: typical abdominal pain, elevation of serum lipase level at three times the upper limit of normal, or evidence of pancreatitis on cross-sectional imaging [18].

**Figure 1:**
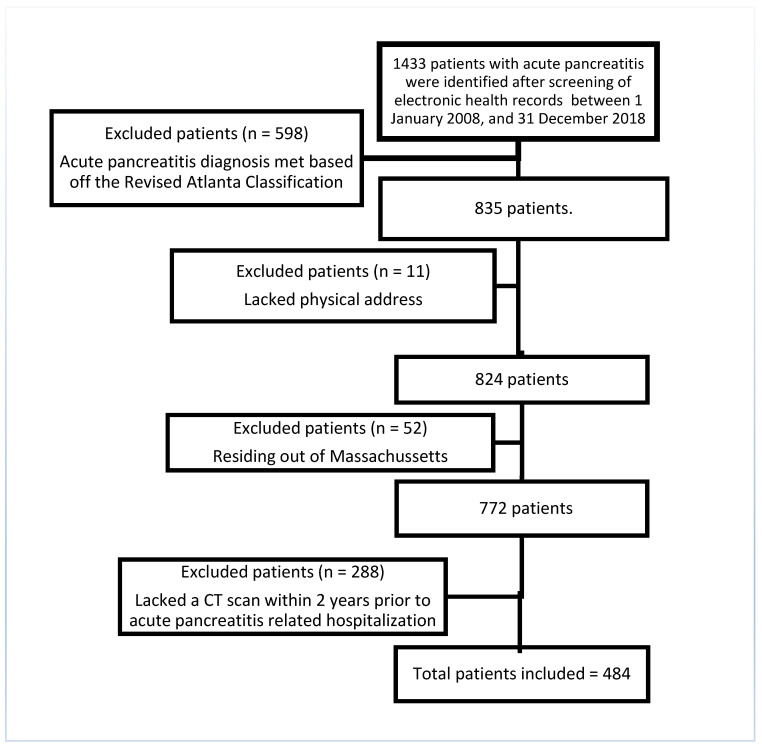
Flow diagram with study population and exclusion criteria.

Exclusions included patients who (1) lacked physical address (required for geospatial coding) and resided outside of Massachusetts and (2) who lacked a CT scan within 2 years prior to acute pancreatitis-related hospitalization (required for radiological features) (Figure 1).

### 2.3. Baseline Demographic Assessment

We reviewed the EHR to collect comprehensive data, including demographic, clinical, laboratory, radiological, and treatment information for patients with acute pancreatitis [19,20]. The demographic characteristics considered for each patient included age, sex, race and ethnicity, active smoking or alcohol use, etiology of acute pancreatitis, insurance type, and address. To accurately locate each patient, we identified and geocoded their local street address. Comorbidity burden was quantified using the Charlson comorbidity index (CCI), which considered pre-existing conditions such as diabetes, kidney disease, pulmonary, and cardiovascular comorbidities [21]. The severity at presentation was assessed using the BISAP score, a scoring system that utilizes blood urea nitrogen, impaired mental status, systemic inflammatory response syndrome (SIRS), age, and the presence of a pleural effusion to predict in-hospital mortality of acute pancreatitis [22,23].

The severity of acute pancreatitis was based on the Revised Atlanta classification and graded as mild acute pancreatitis (no local or systemic complications), moderately severe acute pancreatitis (transient organ failure, local complications, or exacerbation of co-morbid disease), and severe acute pancreatitis (characterized by persistent (>48 h) organ failure) [18]. We also collected data on patient outcomes, including all acute pancreatitis recurrences, the length of hospital stay (LOS), the magnitude of pain upon presentation and discharge quantified by visual analog scale (VAS), opioid use quantified through morphine equivalent units (MME) required during the entire hospitalization, and the presence of local complications such as peripancreatic fluid collections, pancreatic necrosis, and venous thrombosis. Additionally, we recorded systemic complications, including renal failure, respiratory failure, bacteremia, and sepsis. We recorded the disposition of patients, including discharges to extended care facilities, readmissions within a year, and one-year mortality. Furthermore, we collected data on extra-pancreatic complications such as alcohol withdrawal syndrome, gastrointestinal bleeding, and delirium.

### 2.4. Geocoding and Social Vulnerability Index

**Figure 2:**
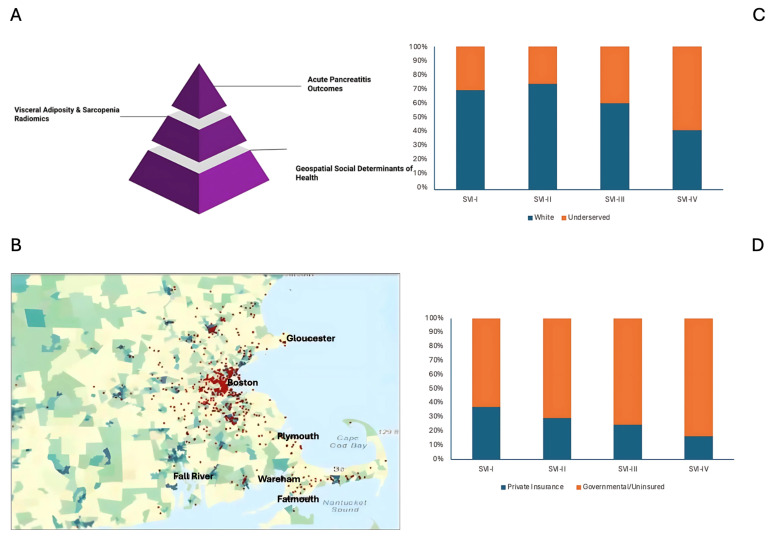
(**A**) Study model describing correlation among geospatial social determinants of health, radiological features, and clinical outcomes of acute pancreatitis. (**B**) Geocoded acute pancreatitis patients cared for at tertiary center in Boston. (**C**) Distribution of Non-Hispanic White as compared to underserved ethnoracial groups. (**D**) Distribution of acute pancreatitis patients with private insurance as compared to those lacking or with governmental insurance.

The physical addresses of acute pancreatitis patients hospitalized at our center in Massachusetts between 1 January 2008–31 December 2021 were geocoded through ArcGIS Pro 2.7.0 (ESRI, Red-lands, CA, USA), (Figure 2A,B). We then combined these locations with the SVI metrics of the census tracts in which they were located [16]. Developed and published by the CDC, the SVI assigns each tract a score ranked on a percentile scale from 0 to 1, 1 indicating the highest vulnerability. SVI values were categorized into four quartiles (I–IV; IV representing the region of highest social vulnerability). This tool evaluates four thematic subdomains: (1) socioeconomic status, (2) household composition and disability, (3) minority status and language, and (4) housing type and transportation, encompassing a total of 16 social attributes.

### 2.5. Radiological Features Assessment

Patients underwent abdominal CT with a 64- or 128-MDCT system (LightSpeed VCT, GE Healthcare; Aquilion, Toshiba America Medical Systems). The tube voltage was 120 kVp, and the tube current was determined by the automatic exposure control.

**Figure 3:**
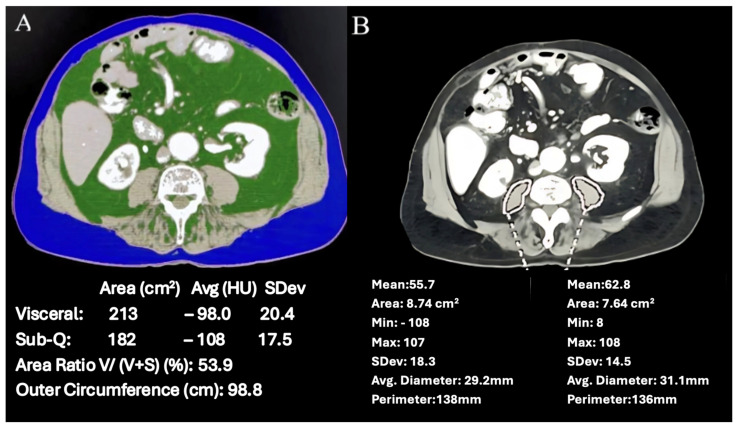
(**A**) Adiposity indices (visceral and subcutaneous fat (cm^2^) and the visceral/total adiposity ratio (VA/TA)) measurement. (**B**) Psoas muscle Hounsfield unit average calculation (HUAC) measurement.

We utilized commercial analysis software (Aquarius iNtuition v4.4.12R, TeraRecon, Durham, NC, USA) to quantify adiposity indices and the psoas muscle attenuation coefficient. Adiposity indices included visceral adiposity (VA), subcutaneous adiposity (SA), and the ratio of VA to total adiposity (VA/TA). Axial CT images of each patient were imported into the TeraRecon workstation, and regions of interest (ROIs) were defined for both visceral and subcutaneous fat compartments using the software’s semi-automated tools at the L3/L4 level. The software then calculated the areas of visceral and subcutaneous fat (cm^2^) and the visceral/total adiposity ratio (VA/TA) (Figure 3A).

Sarcopenia were assessed by measuring the attenuation coefficient of the psoas muscle, termed the Hounsfield unit average calculation (HUAC). Utilizing the TeraRecon software, freehand ROIs were drawn around bilateral psoas muscles on axial images at the L4 level. Subsequently, the area (cm^2^) and the mean density (HU) were recorded for each muscle. The HUAC, a weighted average of the muscle attenuation, accounting for the area of both the right and left psoas muscles was then calculated: HUAC = (right area × density) + (left area × density)/total area (Figure 3B). A lower HUAC indicates less muscle mass or sarcopenia. Levels to determine sarcopenia vary between many publications with the mean numerical numbers ranging from 26–38 [24,25]. As this differs between gender, race, geography, and population, we decided to use study participants as their own controls.

Two outcomes were assessed. The primary outcome was to investigate the association between radiological features and social vulnerability index (SVI) quartiles, specifically VA, SA, VA/TA ratios, and sarcopenia in patients in various SVI quartiles. The secondary outcome included the association between radiological features and clinical outcomes in acute pancreatitis, such as severity of acute pancreatitis.

### 2.6. Statistical Analysis

Categorical data were expressed as proportions, and continuous variables as medians with interquartile ranges (IQR). Chi-square tests evaluated statistical differences in categorical variables, and Kruskal–Wallis tests assessed continuous variables. SVI quartile association with radiologic variables was analyzed using linear regression. For acute pancreatitis severity association with radiologic variables, we performed ordinal logistic regression. Significant associations were further explored to determine the relationship between SVI subdomains, presented as odds ratios (OR). Statistical analysis used STATA software (StataCorp LLC, Version 17.0, College Station, TX, USA), with *p* < 0.05 indicating significance.

## 3. Results

### 3.1. Demographics

From the geocoded database of 772 patients residing within the state of Massachusetts, the study included a total of 484 patients (median age, 54.2 years (IQR 42.6–64.6 years); 277/484 (57.2%) male, 207/484 (42.8%) female) with available radiological features information. Patients with acute pancreatitis had a median BMI of 26.8 (22.9–30.6). History of alcohol abuse was seen in 203/484 (41.94%) patients and smoking history in 160/484 (33.06%) patients. There were 592 acute pancreatitis-related hospitalization events in the 484 eligible acute pancreatitis patients, for which residential locations were divided into four SVI ranking quartiles: Quartile I (*n* = 162), Quartile II (*n* = 128), Quartile III (*n* = 87), and Quartile IV (*n* = 107). Among higher SVI quartiles or patients from more vulnerable regions, we noted significantly more patients who were ethno-racially underserved (I: 108 (66.7%); II: 89 (69.5%); III: 65 (74.7%); IV: 92 (86.0%); *p* = 0.003; Figure 2C) as well as who were uninsured or reliant on governmental agencies (SVI: 54 (33.8%), II: 37 (29.4%); III: 39 (47.6%); IV: 63 (61.2%); *p* < 0.001; Figure 2D). The patient demographics, radiological features, and acute pancreatitis outcomes across SVI are summarized in Table 1.

### 3.2. Clinical Characteristics

**Figure 4:**
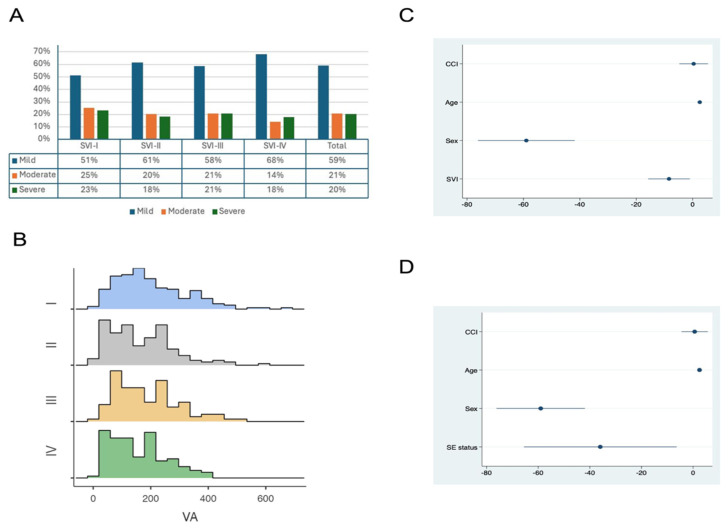
Across SVI quartiles (I–IV, least to most socially vulnerable), distribution of (**A**) acute pancreatitis severity and (**B**) visceral adiposity (VA). After adjustment for age, sex, and comorbidity burden, regression analysis showing association of visceral adiposity with social vulnerability index (SVI) (**C**) and subdomains of socioeconomic status (**D**). CCI- Charlson comorbidity index; SVI- social vulnerability index.

Among 592 acute pancreatitis-related hospitalizations, the severity of acute pancreatitis was categorized as mild in 350/592 cases (59.1%), moderate in 122/592 (20.6%) cases, and severe in 120/592 (20.3%) cases. The predominant etiology was biliary (155/592 (26.2%)), followed by alcohol use (135/592 (22.8%)) and idiopathic acute pancreatitis (131/592 (22.1%)). There were no significant differences in severity of acute pancreatitis across SVI groups (*p* = 0.10). (Figure 4A) Recurrent acute pancreatitis occurred in 75/484 (15.50%) patients and lacked significant association with SVI (I:20/162 (12.35%), II:24/128 (18.75%), III:13/87 (14.94%), IV:18/107 (16.82%)). The median (IQR) LOS was 5 (3–8.9) days with no significant differences among SVI groups (*p* = 0.59). ICU stay was required for 118/592 acute pancreatitis hospitalizations (19.9%), with no significant differences among SVI groups (*p* = 0.50). Acute kidney injury (AKI) requiring dialysis occurred in 50/592 hospitalizations (8.4%), with no significant differences among SVI groups (*p* = 0.36). Delirium occurred in 30/592 hospitalizations (5.1%), with no significant differences among SVI groups (*p* = 0.22).

### 3.3. Radiological Features

The median VA was 176 (IQR 100–251). There was a significant difference across SVI groups: 188.5 (IQR 119–275) in SVI-I, 165.5 (IQR 82.9–243.5) in SVI-II, 185 (IQR 108–251) in SVI-III, and 151 (IQR 88.3–216) in SVI-IV (*p* = 0.03). (Figure 4B) The VA/TA ratio median was 43.5 (IQR 32.3–55.3), with significant differences among SVI groups: 43.8 (IQR 34.6–56.4) in SVI-I, 43.5 (IQR 30.5–55.3) in SVI-II, 45.0 (IQR 33.9–58) in SVI-III, and 40.4 (27.7–50.8) in SVI-IV (*p* = 0.04). There was no significant association between HUAC and SVI quartiles (Table 1).

Out of the 484 patients with 592 hospitalizations, 409/484 (84.5%) patients had a single hospitalization for acute pancreatitis, with 75/484 (15.5%) patients having multiple admissions for acute pancreatitis. When comparing single vs. recurrent hospitalization patients, there was significantly higher median VA with recurrences (VA-single acute pancreatitis: 149 (77.4–233) vs. VA-recurrent acute pancreatitis: 177 (108–256); *p* = 0.04) but no significant difference with HUAC (*p* = 0.23).

### 3.4. Regression Analysis

The regression analysis examined the relationship between VA, SA, VA/TA ratio, and HUAC with SVI (Table 2 and Table 3). The results indicated a significant negative association between VA and SVI, with a coefficient of −9.0 (*p* = 0.04) after adjustment for age, health care behaviors (i.e., active smoking and drinking), and CCI, suggesting that residence in areas with higher social vulnerability index is linked to lower VA (Figure 4C). Similarly, the ratio of visceral to total adiposity (VA/TA) was negatively associated with SVI (coefficient: −0.84, *p* = 0.19) although lacking significant association after adjusting for age, health care behaviors (i.e., active smoking and drinking), and CCI. SA and HUAC showed no significant association with SVI. Given the significant association of visceral adiposity with SVI, we noted substantial negative impact of one subdomain, socioeconomic status (coefficient: −39.80; *p* = 0.03) (Figure 4D), despite adjustment for covariates (age, active smoking and drinking, and CCI). The link between VA and minority status and language, although initially significant, then lacked significance with VA after controlling for confounders. Patients with more severe acute pancreatitis had significantly higher VA (coefficient 37.02, *p* = 0.001), higher VA/TA ratio (coefficient 4.77, *p* = 0.001), and lower HUAC (coefficient −2.94, *p* = 0.001).

## 4. Discussion

In this study, the geospatial analysis of patients at our center revealed that those from areas with higher social vulnerability, as measured by the SVI, had lower visceral adipose tissue (VA) and lower ratios of visceral adipose tissue to total adipose tissue (VA/TA). Despite this, there was no significant association between higher SVI status and severity or recurrence of acute pancreatitis.

The negative association between VA and SVI was particularly prominent in the subdomain of socioeconomic status (encompasses poverty level, unemployment, housing cost burden, lack of health insurance/no high school education). Once adjusted, the association between adipose tissue measurements and the minority status and language subdomains of the SVI became less pronounced, indicating potential effect modification or confounding factors influencing this relationship. The lower VA in the more socially vulnerable group stands in contrast with other studies utilizing SVI in which higher SVI is typically associated with high proportion of obesity and related cardiometabolic complications [26]. This paradoxical finding in our patient population does not have a precise mechanism and will require further study.

In review of the existing literature, a Korean study utilizing SVI showed that obesity (a surrogate for visceral adiposity) was more prevalent in the less socially vulnerable groups [27]. In contrast, in United States adults, seemingly the opposite holds true. An ecological study in Colorado utilizing SVI found that county-level SVI accounted for 41% of the variability in overweight and obesity prevalence [28]. Patterns of obesity based on the socioeconomic status differ from country to country, and within a country, from region to region. In traditionally high-income countries, such as the United States, UK, Canada, etc., the obesity rate tends to be high in the vulnerable population, whereas in low-income countries, the obesity rate has been found to be higher in people with a higher socioeconomic level. In the Colorado study, the population in question included all counties in the state, including a significant rural sector, limiting the applicability of their conclusions to our study. Our results could be influenced by physical lifestyle and food access differences that we cannot account for in our retrospective study. As such, our findings need replication in a larger, multicentered fashion to allow for greater generalizability.

Association of increased visceral adiposity/sarcopenia and acute pancreatitis severity found in this study is consistent with previous data [10]. Visceral fat is metabolically active and can release pro-inflammatory cytokines, exacerbating pancreatic inflammation. Visceral obesity is known to be associated with lipotoxicity due to the upregulation of unsaturated fatty acids released by the process of lipase-induced visceral fat necrosis [8]. Reduced clearance and fat oxidation in individuals with high visceral adipose tissue result in a proportionally higher concentration of unsaturated fatty acids, which may be a trigger and worsening factor for acute pancreatitis [7]. Sarcopenia, independently, and in combination with obesity as sarcopenic obesity, has been found to increase the risk of mortality for patients with severe acute pancreatitis [11]. Additionally, we also found a higher median VA in patients with recurrent pancreatitis vs. single episodes.

A major strength of this study is a distinctive approach to assessing clinical outcomes in acute pancreatitis patients with well documented clinical information, obtained via manual review of individual medical records to ensure accurate acute pancreatitis diagnosis. Reviewing each patient’s scan enhanced the accuracy of diagnosis and allowed for the correlation of radiological feature parameters, providing deeper insights into disease characteristics. We geocoded each patient’s precise geographic location, a feature typically unavailable in publicly accessible databases due to privacy concerns. SVI additionally presents a composite characteristic to study this patient population allowing integration of multiple social determinants of health into a single measure which can standardize social vulnerability, making it easier to compare across different regions and populations when pursuing larger multicentered studies.

There are several limitations to our study. SVI and corresponding geospatial analysis typically employ census tracts as the unit of measurement, rather than individual patient data [29,30]. The geographical aggregation of acute pancreatitis patients has propensity to predispose this observation to ecological bias, which occurs when assumptions about individuals are made based on group-level data. To ensure internal validity, we compared aggregate geospatial socioeconomic variables with individual-level data, verifying that our findings accurately reflect the real-world conditions of acute pancreatitis patients and their social vulnerabilities. Specifically, areas with higher social vulnerability exhibited greater representation of minorities and a higher proportion of uninsured or federally insured individuals, consistent with established social vulnerability indicators. This study is retrospective in design and performed in a single urban center, limiting its generalizability. Its reliance on participants’ physical addresses fails to capture the homeless population and may introduce some level of bias by making the higher SVI groups healthier than expected [31,32]. The study also lacks details on the extent of alcohol consumption behavior and detailed nutritional assessments. Our selection in psoas muscle attenuation as our imaging marker of sarcopenia and frailty, although validated, does not entirely capture sarcopenia which is a more systemic condition. We should also emphasize that this is not a cohort study, and so a significant portion of mild acute pancreatitis patients are excluded from analysis, as patients with mild acute pancreatitis typically do not undergo CT imaging. Lastly, the wide confidence intervals for the associations between our primary and secondary outcomes likely reflect the impact of our sample size. When reviewing other studies utilizing SVI, these are typically studies that involve much larger population datasets, frequently encompassing thousands to millions of individuals or numerous geographic units [27].

## 5. Conclusions

This study elucidates the complex interplay between obesity, sarcopenia social determinants of health, and acute pancreatitis. We determined that lower visceral adiposity predominates in acute pancreatitis patients from more socially vulnerable areas. Higher visceral adiposity and sarcopenia were also linked to increased acute pancreatitis severity. Despite these associations, when comparing SVI with acute pancreatitis severity and recurrence, no significant differences were detected. These findings underscore the impact of visceral obesity and sarcopenia as possible targets to influence acute pancreatitis outcomes across all social strata. While we recommend considering factors that elevate community SVI scores to optimize discharge planning and follow-up strategies, our results caution against oversimplified applications of SVI in risk reduction efforts or healthcare policy aimed at addressing social determinants of health. Further prospective research is needed to validate these results with the aim of supporting targeted interventions promoting health equity in acute pancreatitis management.

## Figures and Tables

**Table 1:** Patient demographics, radiological features, and acute pancreatitis outcomes across social vulnerability (SVI).

**Patients with Acute Pancreatitis**						
**Parameter**	**Total (*n* = 484)**	**SVI-I (*n* = 162)**	**SVI-II (*n* = 128)**	**SVI-III (*n* = 87)**	**SVI-IV (*n* = 107)**	***p* Value**
Age [median (IQR)]	54.2 (42.6–64.6)	56.9 (43.0–67.9)	53.4 (39.1–66)	56.0 (44.7–61.2)	50.3 (41.1.–61.8)	0.22
Sex [(Female); *n* (%)]	207 (42.8%)	65 (40.1%)	56 (43.8%)	38 (43.7%)	48 (44.9%)	0.87
BMI [median (IQR)]	26.8 (22.9–30.6)	27.5 (22.8–30.7)	25.1 (22.8–29.1)	27.4 (23.1–31.5)	26.8 (22.6–30.8)	0.19
Uninsured/Reliant on Governmental insurance agencies [*n* (%)]	193 (41.0)	54 (33.8)	37 (29.4)	39 (47.6)	63 (61.2)	0.001
Ethno-racially underserved patients [*n* (%)]	354 (73.1)	108 (66.7)	89 (69.5)	65 (74.7)	92 (86.0)	0.003
Alcohol [*n* (%)]	203 (41.94%)	65 (40.12%)	58 (45.31%)	33 (37.93%)	47 (43.93%)	0.71
Smoking [*n* (%)]	160 (33.06%)	46 (28.40%)	41 (32.03%)	29 (33.33%)	44 (41.12%)	0.41
Recurrent acute pancreatitis [*n* (%)]	75 (15.50%)	20 (12.35%)	24 (18.75%)	13 (14.94%)	18 (16.82%)	0.49
Visceral Adiposity [median (IQR)]	176 (100–251)	188.5 (119–275)	165.5 (82.9–243.5)	185 (108–251)	151 (88.3–216)	0.03
Subcutaneous adiposity [median (IQR)]	209.5 (138.5–307)	213 (139–295)	183 (128–293)	209 (148–288)	231 (144–341)	0.27
Visceral/Total Adiposity ratio [median (IQR)]	43.5 (32.3–55.3)	43.8 (34.6–56.4)	43.5 (30.5–55.3)	45.0 (33.9–58)	40.4 (27.7–50.8)	0.04
Hounsfield Unit Average Calculation: Muscle Mass [median (IQR)]	51.3 (44.4–58.9)	50.6 (44.1–57.8)	52.7 (58.7–45.0)	49.3 (43.4–58.9)	53.7 (46.5–60.7)	0.17
**Acute Pancreatitis-Related Hospitalizations**						
**Parameter**	**Total** **(*n* = 592)**	**SVI-I** **(*n* = 193)**	**SVI-II** **(*n* =1 58)**	**SVI-III** **(*n* = 106)**	**SVI-IV** **(*n* = 135)**	***p* Value**
Etiology [*n* (%)]	Biliary: 155 (26.2) Alcohol: 135 (22.8) Idiopathic: 131 (22.1) Anatomic: 124 (21.0) Iatrogenic: 18 (3.0) Drug: 11 (1.9) HTG: 10 (1.7) Genetic: 8 (1.3)	Biliary: 46 (23.3) Alcohol: 37 (19.2) Idiopathic: 37 (17.6) Anatomic: 50 (25.9) Iatrogenic: 7 (1.6) Drug: 6 (2.1) HTG: 6 (3.1) Genetic: 4 (0.5)	Biliary: 47 (29.8) Alcohol: 38 (24.1) Idiopathic: 38 (24.1) Anatomic: 25 (15.8) Iatrogenic: 7 (4.4) Drug: 3 (3.2) HTG: 2 (1.3) Genetic: 0 (0.0)	Biliary: 31 (29.3) Alcohol: 27 (25.5) Idiopathic: 20 (18.9) Anatomic: 23 (21.7) Iatrogenic: 1 (0.9) Drug: 1 (0.9) HTG: 1 (0.9) Genetic: 2 (1.9)	Biliary: 32 (23.7) Alcohol: 33 (24.4) Idiopathic: 36 (26.7) Anatomic: 26 (19.3) Iatrogenic: 4 (3,0) Drug: 1 (0.7) HTG: 1 (0.7) Genetic: 2 (1.5)	0.35
Acute Pancreatitis Severity [*n* (%)]	Mild: 350 (59.1) Mod: 122 (20.6) Sev: 120 (20.3)	Mild: 99 (51.3) Mod: 49 (25.4) Sev: 45 (23.3)	Mild: 97 (61.4) Mod: 32 (20.2) Sev: 29 (18.4)	Mild: 62 (58.5) Mod: 22 (20.8) Sev: 22 (20.8)	Mild: 92 (68.1) Mod: 19 (14.1) Sev: 24 (17.8)	0.10
Length of Stay [median (IQR)]	5 (3–8.9)	5 (3–9)	5 (3–8)	4 (3–11)	4 (3–8)	0.59
ICU Stay [*n* (%)]	118 (19.9)	45 (23.3)	27 (17.1)	21 (19.8)	25 (18.5)	0.50
AKI requiring dialysis [*n* (%)]	50 (8.4)	19 (9.8)	10 (6.3)	12 (11.3)	9 (6.7)	0.36
Delirium [*n* (%)]	30 (5.1)	10 (5.2)	12 (7.6)	5 (4.7)	3 (2.2)	0.22

HTG: Hypertriglyceridemia, AKI: Acute kidney injury.

**Table 2:** Association of visceral adiposity: (VA) and Hounsfield unit average calculation (HUAC) with social vulnerability index (SVI) using unadjusted and adjusted regression analysis.

	Social Vulnerability Index			
Parameter	Coefficient	*p* Value	Adjusted Coefficient ^#^	*p* Value
VA	−11.68 [−20.41–−2.94]	0.009	−9.0 [−17.95–0.05]	0.04
SA	9.11 [95%CI: −1.11–19.35]	0.081	-	
VA/TA Ratio	−1.53 [95%CI: −2.75–0.30]	0.01	−0.84 [95%CI: −2.11–0.42]	0.19
HUAC	0.34 [95%CI: −0.55–1.23]	0.45	-	-

adjusted by age, health care behaviors—smoking and alcohol use, and comorbidity burden quantified by Charlson comorbidity index (CCI).

**Table 3:** Regression analysis investigating visceral adiposity across social vulnerability index (SVI) subdomains.

Parameter	Coefficient	*p* Value	Adjusted Coefficient	*p* Value
Socioeconomic status	−51.90 [95%CI: −86.89–−16.91]	0.004	−39.78 [95%CI: −75.88–−3.70]	0.03
Household composition	−15.86 [95%CI: −47.67–15.94]	0.33	−16.90 [95%CI: −49.02–15.21]	0.30
Minority status and language	−48.22 [95%CI: −84.80–−11.64]	0.01	−32.84 [−71.04–5.35]	0.09
Housing type and transportation	−33.86 [95%CI: −70.80–3.07]	0.07	−33.95 [−71.50–3.60]	0.08

adjusted by age, sex, and comorbidity burden quantified by Charlson comorbidity index (CCI).

## Data Availability

Research data can be made available upon request of the corresponding author.

## Abbreviations

The following abbreviations are used in this manuscript:

BMI	Body mass index
CT	Computed tomography scans
(CDC)’s	Centers for Disease Control and Prevention
CCI	Charlson comorbidity index
EHR	Electronic health records
HUAC	Hounsfield unit average calculation
LOS	Length of hospital stay
MME	Morphine equivalent units
VA/TA	Ratio of visceral to total adiposity
SVI	Social vulnerability index
SA	Subcutaneous adiposity
SIRS	Systemic inflammatory response syndrome
VA	Visceral adiposity
VAS	Visual analog scale
